# Insyght: navigating amongst abundant homologues, syntenies and gene functional annotations in bacteria, it's that symbol!

**DOI:** 10.1093/nar/gku867

**Published:** 2014-09-23

**Authors:** Thomas Lacroix, Valentin Loux, Annie Gendrault, Mark Hoebeke, Jean-François Gibrat

**Affiliations:** 1 INRA, UR 1077 Mathématique Informatique et Génome, 78352 Jouy-en-Josas, France; 2 CNRS, UPMC, FR2424, ABiMS, Station Biologique, 29680 Roscoff, France

## Abstract

High-throughput techniques have considerably increased the potential of comparative genomics whilst simultaneously posing many new challenges. One of those challenges involves efficiently mining the large amount of data produced and exploring the landscape of both conserved and idiosyncratic genomic regions across multiple genomes. Domains of application of these analyses are diverse: identification of evolutionary events, inference of gene functions, detection of niche-specific genes or phylogenetic profiling. Insyght is a comparative genomic visualization tool that combines three complementary displays: (i) a table for thoroughly browsing amongst homologues, (ii) a comparator of orthologue functional annotations and (iii) a genomic organization view designed to improve the legibility of rearrangements and distinctive loci. The latter display combines symbolic and proportional graphical paradigms. Synchronized navigation across multiple species and interoperability between the views are core features of Insyght. A gene filter mechanism is provided that helps the user to build a biologically relevant gene set according to multiple criteria such as presence/absence of homologues and/or various annotations. We illustrate the use of Insyght with scenarios. Currently, only Bacteria and Archaea are supported. A public instance is available at http://genome.jouy.inra.fr/Insyght. The tool is freely downloadable for private data set analysis.

## INTRODUCTION

Genomic regions undergo various types of rearrangement at micro and macro scales due to different evolutionary processes. This leads to translocations, duplication, fusion, fission, loss or inversion (1). Those events participate in conferring the uniqueness of each species or individuals (2,3). From a multi-species comparison perspective, each genome can be seen as a succession of regions that are either distinctive or conserved at various degrees. Conserved synteny (or shared synteny) refers to the co-localization of homologous loci across different species. If in addition the ordering of the genes is preserved, the conserved synteny is then labelled as collinear. Often, a variety of terms such as ‘synteny’ or ‘synteny block’ are used in lieu of conserved or collinear synteny (4).

High-throughput sequencing technologies have become commonplace and biologists need tools that assist them in annotating gene functions quickly and accurately at a genome-wide scale. Together with sequence similarity, gene neighbourhood conservation and phylogenetic profiles provide important clues to identify orthologous genes or infer gene functions (5,6). Conservation in the ordering of genes can help in assigning functions for a train of genes at once or providing clues for hypothetical proteins (7,8). Moreover, shared synteny may indicate a relationship between gene products such as protein–protein interaction (9) or functional coupling (10,11). Transcriptional activity has also been correlated to conserved synteny in expression pattern and transcriptional regulation studies (12,13). Several annotations platforms consider shared synteny as the cornerstone in their analysis strategy (14–19).

Conservation of genes across species can also hint to valuable information regarding broader biological issues such as the evolutionary history of a particular genome (20–22), positive selection arising from evolutionary constraints (23), rearrangement mechanisms (24–26) or regions with critical functional activity (27). On the other hand, distinctive genomic regions and niche-specific genes are crucial in understanding what makes each species and individual different.

## Visualization methods for synteny and homology relationships

**Figure 1.**
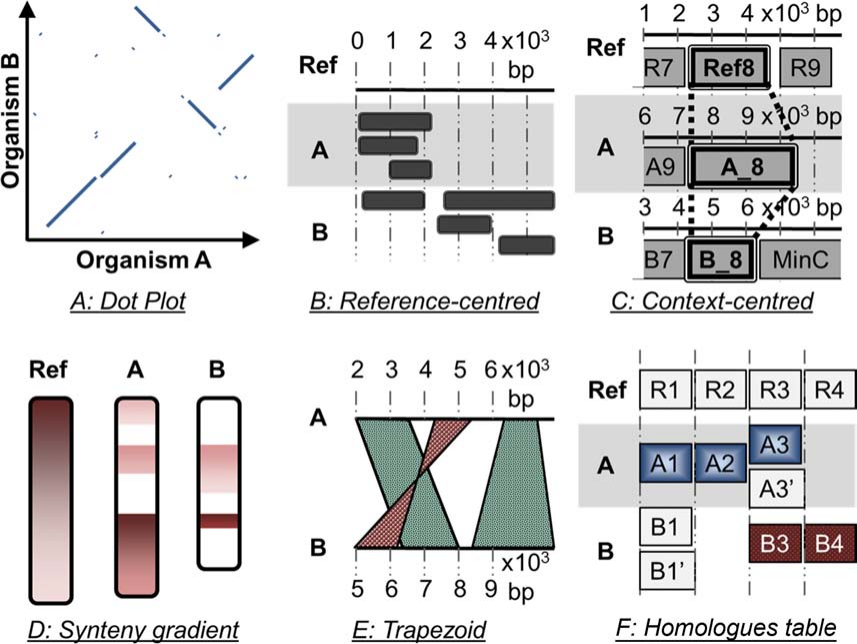
The different graphical paradigms used for the visualization of syntenies and homologies. (**A**) Dot Plot. (**B**) Mapping of conserved features onto a reference region (reference-centred view). (**C**) Genomic contexts view centred on the reference gene Ref_8 and its homologues. (**D**) Synteny gradient view (banded ideograms). (**E**) Parallel linked track or trapezoid view. (F) Table chart; the cell background is coloured according to synteny.

Graphical representations help in comprehending complex concepts that are not easily grasped by the human mind. They open possibilities for reasoning under different perspectives and therefore assist biologists during their decision-making processes. Many tools have been designed for the purpose of exploring conserved synteny or homology (Supplementary Table S1), and innovative graphical displays have been proposed (28,29) such as the dot plot (Figure 1A), the reference-centred view or block track (Figure 1B), the genomic context-centred view (Figure 1C), the chromosome painting or banded ideograms (Figure 1D), the parallel linked track or trapezoid view (Figure 1E) and the symbolic representation (Figure 1F). Supplementary Table S2 summarizes the pros and cons of the different graphical paradigms used for the visualization of syntenies and homologies. Many tools associate two or more types of views to provide an interconnected and comprehensive set of displays that compensate for each other shortcoming. This allows the user to navigate seamlessly amongst different scales and perform different types of analysis. However, there is still room for improvement and a number of challenges remain, for example, (i) providing the user with a clear detection of the rearrangements that are both scattered across the genomes and occur at different scales, (ii) emphasizing the non-homologous genomic regions located amidst those rearrangements (29) and (iii) designing a seamless navigation amongst a large collection of data and heterogeneous factors: the genomic base pair coordinate system, multiple genomes to compare, multiple homologues per comparison and multiple annotations per gene.

## MATERIALS AND METHODS

### Database and pipeline

Data are stored in a PostgreSQL relational database. The database contains three categories of data: (i) primary data such as genomic annotations that are extracted from complete genomes files (Genbank or EMBL format), (ii) secondary data corresponding to the cross comparisons, using BLASTp, of all the Coding DNA Sequences (CDS) of the stored bacterial genomes and (iii) tertiary data such as synteny regions computed from the secondary data. Blast alignments are performed at the protein level and results with an *e*-value less than 0.01 are stored in the database. We define two genes as being orthologous if they give rise to a Bi-Directional Best Hit (BDBH) in Basic Local Alignment Search Tool (BLAST) comparisons of the corresponding genomes. More information about the concept of homology and orthology in bioinformatics is available on the website. Syntenies are computed with a dynamic programming algorithm that determines the highest scoring paths amongst the chains of collinear homologues. Small gaps are allowed within the conserved synteny. The scores and penalties are as follows: orthologue: 4; homologue: 2; mismatch: −4; gap creation: −8; gap extension: −2; minimum alignment size: 1; minimum score: 2. Dynamic programming is used in tools such as CYNTENATOR (30), DAGchainer (31), FISH (32), i-ADHoRe (33) or SyMAP (4). Some other tools compute conserved synteny on the basis of multiple genomes simultaneously: Cinteny (34), i-ADHoRe (33) and OrthoClusterDB (35). Therefore, our approach to compute orthology relationships and syntenies is based on established methods; it focuses on pair wise comparisons and Insyght is designed to analyse multiple pairs in concert. As mentioned previously, Insyght makes the assumption that there is one protein product per gene and is therefore not suitable for eukaryotes. Our public instance currently contains 407 bacterial organisms (860 chromosomes and plasmids); this is the largest data set on which the tool has been tested so far. The largest cumulative size for an organism is 9.731 Mb (Burkholderia xenovorans LB400).

### Web application

Due attention has been paid to performance during the development of Insyght. Start-up time and most loading times take a few seconds even at the whole genome scale and for multiple comparisons. We use Asynchronous JavaScript and XML (AJAX) technology to minimize data transfer between server and clients, send simultaneous server requests and transfer most of the processing load on the client side. The graphical rendering uses the HTML5 canvas element, which is supported by default on all modern web browsers. The web application is designed so that its performance depends little on an increase of the overall data set. The user experience is quite comparable to stand alone tool with regard to performance and functionality whilst keeping the benefits of web applications: no installation or maintenance, seamless updates and ease of sharing across the web. Browser history support is provided for all tabs as well as the pages within the tabs. History for the navigation amongst the symbols within a comparison is not supported though.

## RESULTS

Insyght proposes a new way to navigate amongst syntenies, homologies and gene functional annotations. This section describes the three complementary displays available in Insyght and highlights what novelties they offer. Insyght does not support organisms with alternative splicing mechanisms at the moment and is therefore only suitable for the analysis of Bacteria and Archaea.

### The genomic organization view: combining methods to improve the visualization of genomic contexts

**Figure 2.**
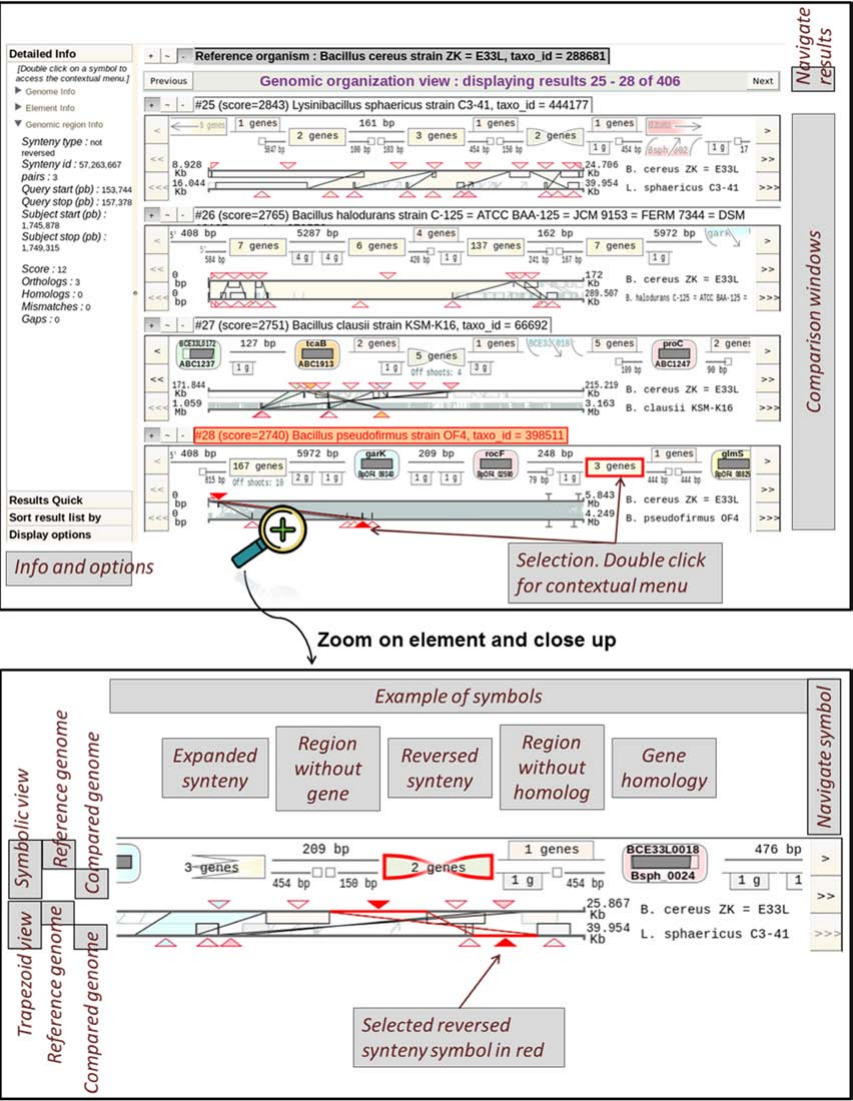
Overview and close-up of the genomic organization view. The overview figure at the top shows that this view is organized into two different parts: on the right side, the comparison results are stacked up on top of each other, each within its own window. On the left side, different stack panels such as « Detailed info », « Result quick navigation », « Sort result list by » or « Display options » provide the user with additional information and functionalities. Each symbol can be selected by clicking on it. Upon selection, both the symbolic and trapezoid views are highlighted in red, and additional information is displayed on the left panel. A contextual menu pops up when the user double clicks on the graphical display and provides contextual options. The figure at the bottom shows a zoom and close-up on a result window. There are two main parts per window: the upper part depicts symbols representing the different genomic annotations, whilst the lower part consists of the trapezoid view. A triangular marker on the trapezoid view indicates the genomic location of each symbol. In both views, the representation for the reference genome is displayed at the top, the representation for the compared genome at the bottom and homologous region span over both top and bottom. Example of symbols: a gene homology symbol is comprised of the reference gene name at the top, a proportional display of the sequence alignment in the middle and the name of the target gene at the bottom. The symbol for conserved synteny is a rectangle or a bowtie if the synteny is reversed. The symbol for a non-homologous genomic region looks either like a line when it has no gene or like a rectangle when it contains genes. Non-homologous genomic regions for the compared genome are represented at half size surrounding the bottom part of each homologous symbol. Users can navigate amongst the annotation symbols either downstream or upstream, or zoom onto a specific region by dragging the mouse lengthwise along the genomic scaffolds.

Insyght proposes a new way to explore the landscape of conserved and idiosyncratic genomic regions across multiple pair wise comparisons. Its unique display is based on the association of the symbolic and the trapezoid graphical paradigms (Figure 2). We expanded the convention of the symbolic paradigm to represent not only homologous genes but also conserved syntenies and non-homologous genomic regions. Therefore the user can browse and interact with a variety of symbols that constitute the chain of annotation events. The symbols are tightly integrated with a display representing the same annotation events drawn proportionally according to their genomic positions and joined up by trapezoids if they are homologous (see ‘trapezoid view’ in the ‘zoom and close-up’ section of Figure 2). The symbols provide legibility whilst the proportional display simultaneously allows grasping genomic locations and complex rearrangements scattered across the genomes and occurring at different scales. Combining the symbolic and proportional representations is a variation of the concept of nonlinear views (36) where the visualization is distorted to highlight the region of interest but still provides the user with all the contextual data. Symbols are ordered according to their start position on the reference genome and the user has the possibility to browse amongst them. The resulting display for the reference genome appears as a succession of homologous symbols followed by non-homologous symbols. This cyclic partitioning contributes to a better legibility of the genomic rearrangements and idiosyncrasies, i.e. genomic regions specific of a particular genome. The representation of genomic regions without homologue on the compared genomes is more challenging, as their locations may appear scattered. We choose to represent them at half scale surrounding the bottom part of the homologous regions as shown in Figure 2 (in the ‘zoom and close-up’ section, see the ‘compared genome’ display of the ‘symbolic view’). From the perspective of the reference genome, a gene or subset of genes within a shared synteny may sometimes have multiple homologous copies at different locations in the compared genome (due to paralogues or duplication of the synteny region). A menu below the symbolic representation is shown whenever such offshoot events occur allowing the user to browse amongst them. The chromosomes and plasmids of an organism are displayed consecutively so that the reorganization between different elements is visible at once. The display can be scaled to a region of interest by dragging the mouse either on the reference or the compared genome. Navigation and zoom across the stacked compared genomes can either be dissociated or synchronized at the discretion of the user. This option offers the possibility to define a common query genomic region and compare the results simultaneously. Additional information about these functionalities is available on the website.

### The orthologues table: exhaustive browsing of arbitrary gene sets

**Figure 3.**
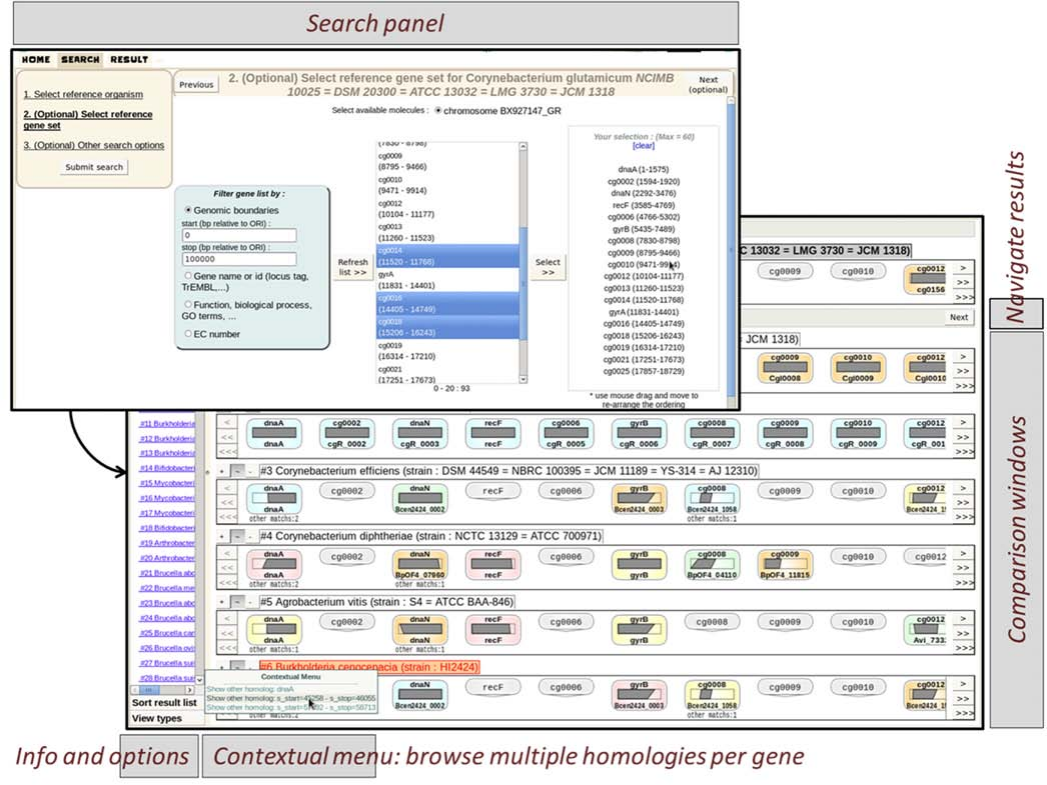
A typical search tab and homologues browsing view. The search panel allows for any combination of genes from the same organism. To facilitate the retrieval of genes of interest, users can perform searches with criteria such as genomic location, name, functional categories, biological processes and EC number (see Supplementary Figure S1). In the homologue browsing view, each column represents a gene from the user-constituted gene set and each row represents a compared organism. The presence or absence of homologue is shown in each cell. If multiple compared genes are homologous to a given reference gene, the user can browse amongst them. The genes are background-coloured according to which synteny they belong to.

We also have implemented a view dedicated to effectively inspecting a large number of orthologues and pinpoint genes with no orthologue in a given species. The display looks like a comparison table where columns are the selected genes and rows are the result species (Figure 3). Multiple overlapping homologies such as duplications/paralogues are stacked in one cell and displayed as ‘offshoots’. The background of genes is coloured according to the synteny they belong to. A thorough analysis of orthologues contributes to minimize shortcomings during the annotation process (37). Although most of the evolutionary relationships between genes displayed by Insyght are ‘orthologues’, some are ‘paralogues’ and others correspond to genes of the reference genome that have several ‘homologues’ in the compared genome (see the database and pipeline section). For the sake of simplicity, we will use the general term ‘homologue’ to describe all evolutionary relationships thereafter. The user can either transfer genes from the genomic organization view or freely build up a combination of any genes from the same organism using the ‘Search’ tab. To facilitate the retrieval of genes of interest and allow phylogenetic profiling, different types of filter have been developed (Supplementary Figure S1); some are based on the intrinsic properties of the gene (genomic location, presence of domains or motifs), others on functional annotations (identifiers, functions, GO terms, EC number, cross-reference to public databanks such as EMBL or Uniprot) or, again, on the presence or absence of homologues in a given set of species. An unlimited number of filters can be combined together with either the operator AND (intersection) or OR (union). Therefore the users have the ability to formulate relevant biological questions, such as finding genes in species A that have homologues in species B and/or C but not D and/or E and that match a few particular functions or biological processes. When selecting a taxonomic node containing multiple organisms, the user has the possibility to browse the different combinations of core/dispensable gene set. A reference genome is chosen by default and can be changed as well as the genomes to assert for the presence or absence of homologues.

### The annotations comparator: a shift in perspective

**Figure 4.**
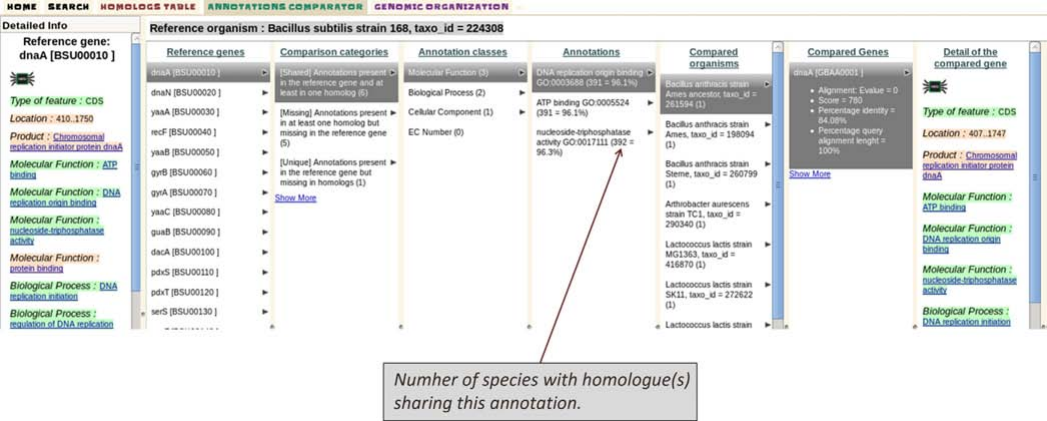
The annotations comparator. Functional annotations amongst homologues are classified into three main categories: shared, missing and unique. The figure shows all the subcategories: the functional annotation classes (molecular function, biological process, cellular component, EC number), the ontologies assigned to the gene(s), the list of compared organisms, the homologous genes, a summary of the sequence alignment and the detailed information of the compared gene. The number of items that belong to a given subcategory is shown within parentheses. For example, in parentheses next to a given reference gene functional annotation is the number of species with homologue(s) sharing this annotation. The percentage value shown is a ratio to the total number of organisms compared.

A list of reference genes can be transferred from the other views or build via the ‘Search tab’ to be visualized with the annotations comparator. This view compares the functional annotations of the reference gene and its homologues and classifies the annotations into three categories: (i) [Shared] annotations present in the reference gene and at least in one homologue, (ii) [Missing] annotations present in at least one homologue but missing in the reference gene and (iii) [Unique] annotations present in the reference gene but missing in homologues. By selecting one of these categories, the user has the possibility to navigate in the following cascading subcategories: the functional annotation classes (molecular function, biological process, cellular component, EC number), the functional annotations assigned to the gene(s), the list of compared organisms, the homologous genes, a summary of the sequence alignment and detailed information about the compared gene (see Figure 4). When this last subcategory is shown, the similarities and discrepancies between the reference gene functional annotations and its homologue are highlighted in green and red, respectively. The number of items that belong to a given subcategory is shown within parentheses. For example, in parentheses next to a given reference gene functional annotation is the number of species with homologue(s) sharing this annotation (Figure 4). This number is an indication of the degree of commonality of a given annotation amongst homologues. The annotations comparator relies mostly on functional annotations based on a controlled vocabulary such as gene ontology (i.e. molecular function, biological process); it is less relevant for fields that are typically more heterogeneous like product. The set of compared organisms can be restricted to a subset of the taxonomy. Homologues can be filtered according to *e*-value, minimum percentage identity, minimum percentage query alignment length and whether or not they correspond to orthologues. Some existing tools offer the possibility to query a set of biological annotations and generate the corresponding list of homologues; this functionality is referred to as ‘annotation-centred’ in the column ‘Functional annotations comparator’ of the Supplementary Table S1. To the best of our knowledge, Insyght is the only tool so far to also propose a feature that classifies the annotations of a given gene and its homologues depending on their degree of commonality.

In the three views, double-clicking on a symbol or reference gene results in a menu popping-up. Examples of commands available from this menu consist in transferring gene(s) to another view, expanding the genes within a synteny or a genomic region, zooming on a specific chromosome or plasmid, synchronizing the displays on a given gene, exporting the gene sequence, exporting the whole table as a comma separated file… Other functionalities, such as synchronized navigation and zooming across multiple species, excluding/featuring compared genomes, resizing the views, sorting the result list according to various criteria, bookmarking/sharing views, etc., are provided outside the contextual menu. A comprehensive documentation about Insyght usage is available on the website.

## DISCUSSION

Insyght can be used either for assigning gene function to newly sequenced genomes or whilst analysing already annotated organisms. In this section, we discuss an example use case followed by the conclusion.

### Comparative genomics analysis of already annotated genomes

Analysis of niche-specific genes or genes of the core genome in the context of closely or distantly related species is one approach for finding candidates related to a given phenotypic or phylogenetic trait (38,39). We used Insyght to analyse the dispensable genome of *Enterococcus faecalis* V583 and find gene candidates in relation to pathogenicity. *E. faecalis* is part of the commensal gut microbiota in humans and animals and has been identified as a public health threat responsible for severe nosocomial infections such as urinary tract infection, bacteremia or endocarditis (40,41). Its genome harbours a putative pathogenic island, seven prophage-related elements and other mobile elements that support *E. faecalis* propensity for horizontal gene transfer (42,43). Our database contains the four complete genomes of *E. faecalis* isolates from human source to date, of which three are pathogenic to human according to the Genomes OnLine Database (44).

The first step in the analysis was performed using Insyght search ability and the orthologues table view. The dispensable genome of V583 was retrieved by searching for different combination of presence/absence of homologues with the 62, OG1RF and Symbioflor1 strains. Supplementary Table S3 contains 20 loci of interest regarding horizontal genomic transfers that have been mentioned in various studies (42,45–47). The number of genes from the dispensable genome that belong to those loci is reported as well as the *P*-value calculated according to the binomial law. This result shows that the distribution is very significantly biased towards those loci. Genes from those loci were easy to spot because they stood out as clusters of neighbour genes and made up for 70% of the overall dispensable gene set. The orthologues table view was then used to search the dispensable gene set for collinear syntenies that were conserved in other opportunistic pathogens. Three loci stood out: EF_1875-EF_1879/EF_2277-EF_2281 (Supplementary Figure S2) that appear to be duplicated and EF_2270-EF_2272. EF_1875-EF_1879/EF_2277-EF_2281 are conserved in our data set amongst 24 firmicutes, all of which share common pathogenic traits such as infection of deep tissues/organs and blood stream (Supplementary Table S4). Using the binomial law, we estimate the *P*-value of this event to be 3.8E−7 as 54% of the 203 firmicutes in our data set are pathogens. With regard to EF_2270-EF_2272, this synteny is present within 35 firmicutes pathogenic to human and five non-pathogen species in our data set (Supplementary Table S5) (*P*-value = 5.8E−6).

Subsequently, we used the genomic organization view to analyse the genomic surroundings of those loci across the 24 species in parallel. All the syntenies fall into well-known hotspots for mobile elements in V583: region *efaB5* (EF_1847-EF_1897) and region *vanB* (EF_2240-EF_2351). We noted four homologies that are often found within close vicinity of the collinear syntenies amongst the 24 species: EF_1850, EF_1882, EF_1886 and EF_1895.

We then transferred the genes of interest in the annotations comparator view to get an overview of the functional annotations amongst the homologues. Some genes may constitute interesting candidates for further biological investigation. For example, proteins such as EF_1876/EF_2278 or EF_1877/EF_2279 that seem to be mostly present in pathogenic species and accessible from outside the cellular wall may constitute interesting therapeutic targets (48,49). With regard to EF_2270 and EF_2271, some components of the phosphoenolpyruvate-dependent sugar phosphotransferase system have been linked to pathogenicity in previous studies (50–52). EF_2272 is also of interest as predicted Glucuronyl hydrolase activity may be involved in the infectious process of some pathogenic streptococci (53), more specifically during the adhesion stage to the mammalian cell surface matrix.

### Functional annotation for newly sequenced genomes

When performing functional annotation based on the transfer of annotation from orthologues, each view in Insyght is adapted to a number of tasks at hand: the genomic organization view is convenient for focusing on a few pair wise comparisons with closely and well-annotated genomes, the table is useful to easily browse amongst orthologues from a dozen organisms of interest whilst keeping information on syntenies in sight, and the annotations comparator summarizes the functional annotations of all the orthologues. The ability of transferring genes from one view to the other is particularly handy.

Many studies, see for instance (54–58), have pointed out errors in the functional annotation of numerous genes. The percentage of erroneously annotated genes is estimated to lie between 5 and 40%. There are various causes of errors, but a large number are due to the transfer of functional terms between homologues with low percentage of sequence similarity. With this consideration in mind, the annotations comparator view in Insyght provides the user with the option to adjust the set of compared organisms and the alignment similarity thresholds (*e*-value, minimum percentage identity, minimum percentage query alignment length and BDBH). Also, the local conservation of syntenies between genomes can increase annotator confidence when they need to transfer functional annotations from the genes of one genome to those of the other displaying marginal sequence similarities.

## Conclusion and perspectives

Growth of data in the field of comparative genomic is pushing existing visualization tools to their limits (59). Here, we presented a new tool tailored to the analysis of homologies and syntenies in prokaryotes. To the best of our knowledge, the novelties that Insyght brings forward are: (i) a genomic organization view that associates symbolic and proportional representations which increase the legibility of genome rearrangements and non-homologous genomic regions, and (ii) an annotations comparator that classifies the functional annotations of homologues into three categories depending on their commonality. Moreover, Insyght constitutes an improvement over other existing tools with regard to the interoperability between the views and the possibility to create an arbitrary gene set that can be further explored with the orthologues table view or the annotations comparator. We believe those innovative designs and functionalities will assist biologists in performing fast and efficient data mining of the conserved synteny, homologues and distinctive genomic regions. By downloading the virtual machine, biologists can work on their private sequences and focus on a group of closely related genomes of their choice.

We plan to continue to develop Insyght by integrating more complete prokaryote genomes in our public database and implementing links to complementary tools such as our genome annotation platform, AGMIAL (60).

## AVAILABILITY

Insyght is an open source project under the CeCILL-B licence. The home page of the project is http://genome.jouy.inra.fr/Insyght. To facilitate the analysis of private data, a virtual machine can be downloaded and installed locally. It contains a pre-installed version of the pipeline, the database with a dozen example genomes and the visualization tool. The documentation on how to run the perl scripts of the pipeline to create a user-tailored database and visualize the results is provided. Creating a database of 20–30 organisms takes 1 or 2 days on a laptop Intel Core i7-2620M 2.7Ghz 8 Go RAM. As the number of comparisons grows exponentially with the number of genomes to compare, the insertion of a non-trivial amount of organisms is consuming in terms of computer resources. The schemas for the database and the pipeline are available on the website.

## SUPPLEMENTARY DATA

Supplementary Data are available at NAR Online.

SUPPLEMENTARY DATA
