# Supplementary material for: Insyght: navigating amongst abundant homologues, syntenies and gene functional annotations in bacteria, it's that symbol!
Source: Nucleic Acids Res. 2014 Sep 23;42(21):e162. doi: 10.1093/nar/gku867 (PMC4245967; doi:10.1093/nar/gku867)
Supplement: SUPPLEMENTARY DATA [file supp_42_21_e162__index.html]

Insyght: navigating amongst abundant homologues, syntenies and gene functional annotations in bacteria, it's that symbol! — Insyght: navigating amongst abundant homologues, syntenies and gene functional annotations in bacteria, it's that symbol! — SUPPLEMENTARY DATA 

# Insyght: navigating amongst abundant homologues, syntenies and gene functional annotations in bacteria, it's that symbol!

## SUPPLEMENTARY DATA

**Files in this Data Supplement:**

- SUPPLEMENTARY DATA
- SUPPLEMENTARY DATA
