# Supplementary material for: Insyght: navigating amongst abundant homologues, syntenies and gene functional annotations in bacteria, it's that symbol!
Source: Nucleic Acids Res. 2014 Sep 23;42(21):e162. doi: 10.1093/nar/gku867 (PMC4245967; doi:10.1093/nar/gku867)
Supplement: SUPPLEMENTARY DATA [file supp_gku867_nar-01151-met-n-2014-File006.pdf]

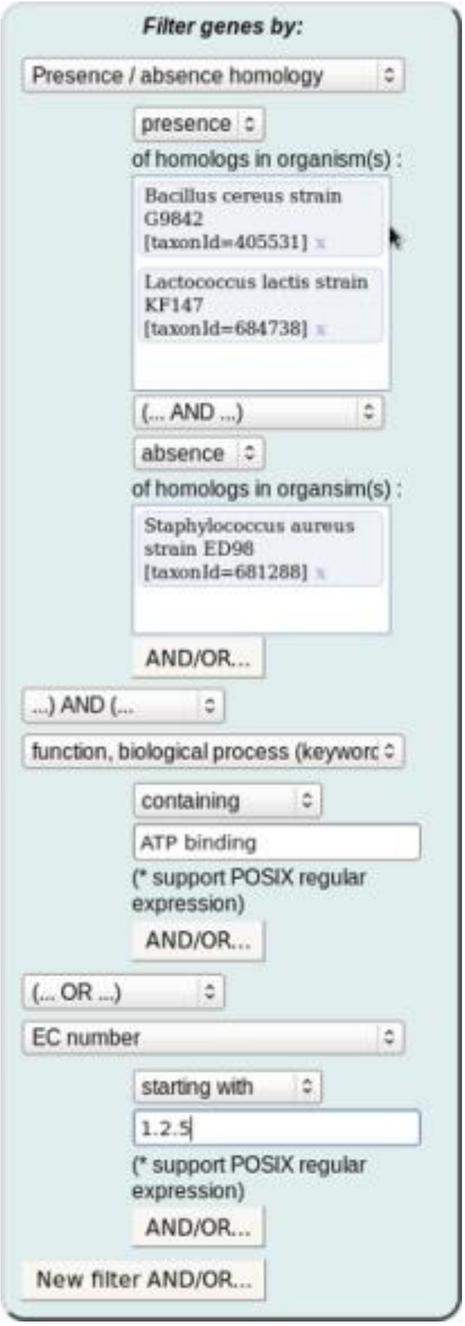

Supporting Figure 1. The filter box. An unlimited number of filters of different types (genomic location, presence / absence of homologs, gene ids, function, biological process, product, cellular component, EC number, type of evidence) can be combined with the operators AND (intersection) or OR (union).

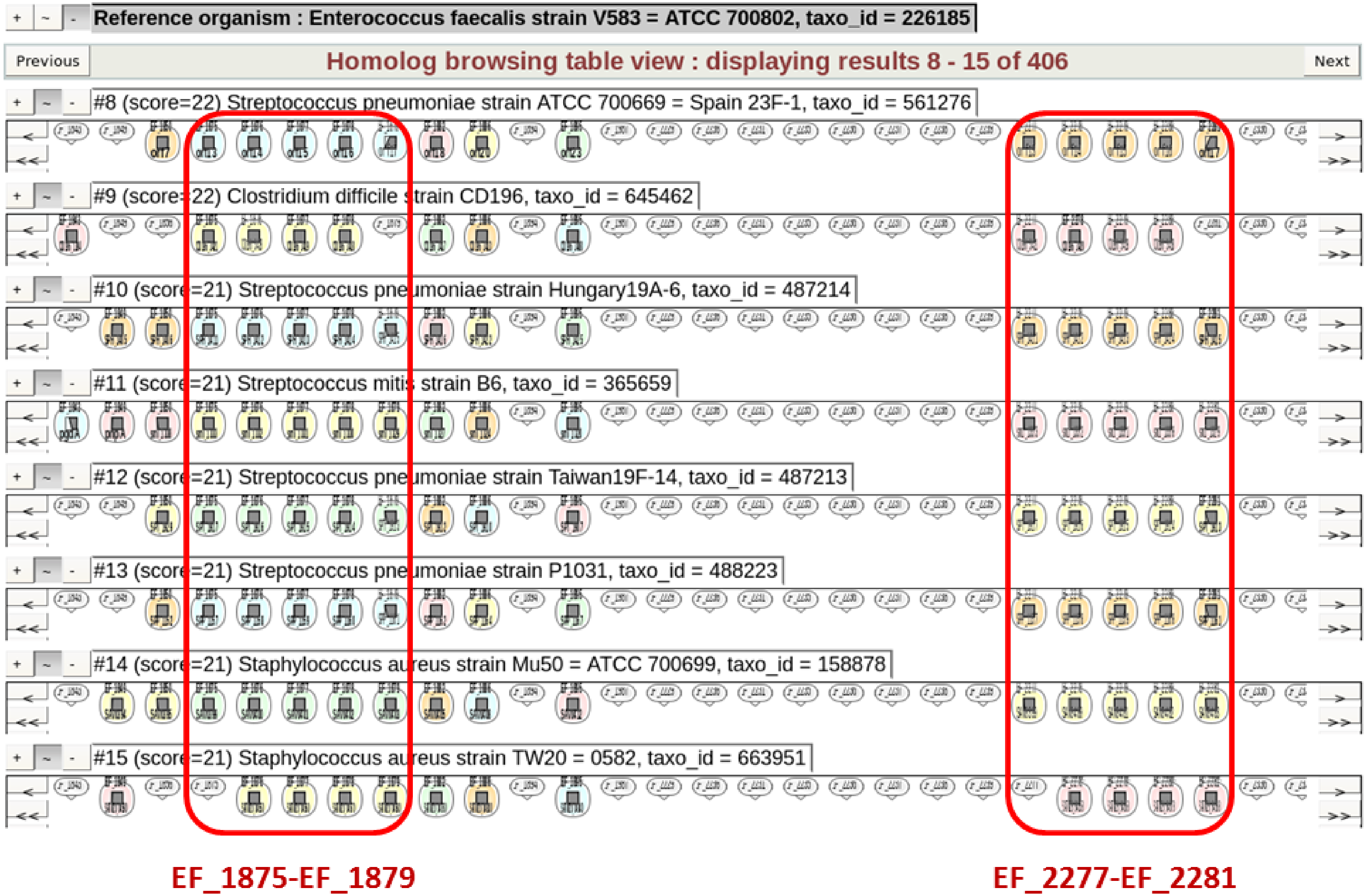
Reference organism : Enterococcus faecalis strain V583 = ATCC 700802, taxo_id = 226185
Previous
Homolog browsing table view : displaying results 8 - 15 of 406
Next
#8 (score=22) Streptococcus pneumoniae strain ATCC 700669 = Spain 23F-1, taxo_id = 561276
#9 (score=22) Clostridium difficile strain CD196, taxo_id = 645462
#10 (score=21) Streptococcus pneumoniae strain Hungary19A-6, taxo_id = 487214
#11 (score=21) Streptococcus mitis strain B6, taxo_id = 365659
#12 (score=21) Streptococcus pneumoniae strain Taiwan19F-14, taxo_id = 487213
#13 (score=21) Streptococcus pneumoniae strain P1031, taxo_id = 488223
#14 (score=21) Staphylococcus aureus strain Mu50 = ATCC 700699, taxo_id = 158878
#15 (score=21) Staphylococcus aureus strain TW20 = 0582, taxo_id = 663951
EF_1875-EF_1879
EF_2277-EF_2281

Supporting figure 2. Example of the homolog table view showing a subset of the dispensable genes set of E. faecalis V583 and their homologs among a few compared species. The two duplicated collinear syntenies EF_1875-EF_1879 and EF_2277-EF_2281 are highlighted in red. They are conserved in our dataset among twenty-four firmicutes, all of which are pathogenic to human (see Supporting Table 4).
