# Supplementary material for: Insyght: navigating amongst abundant homologues, syntenies and gene functional annotations in bacteria, it's that symbol!
Source: Nucleic Acids Res. 2014 Sep 23;42(21):e162. doi: 10.1093/nar/gku867 (PMC4245967; doi:10.1093/nar/gku867)
Supplement: SUPPLEMENTARY DATA [file supp_gku867_nar-01151-met-n-2014-File007.pdf]

Supporting Table 1. Graphical paradigms implemented in 41 synteny and homology visualisation tools; the target taxonomic kingdoms and a few functionalities centred on specific ways to build and compare gene sets are presented as well (columns 9-12)". Regarding the "Symbolic representation" column, a "+" is awarded if the symbols for gene or gene homology are available, another "+" is awarded if the symbolic representation is extended to other genomic regions such as conserved syntenies or genomic region insertions. With regard to the "Filters for phylogenetic profiling" column, a "A" is awarded for tools which offer search functionality based on the presence / absence of homology with a set of compared genomes, a "B" is awarded if multiple filters can be combined with the AND operator (intersection) and the OR operator (union), a "C" is awarded if the presence / absence of homology filter can be combined with other types of filters (genomic data, etc.), a "D" is awarded if the homology criteria can be parameterised, and a "E" is awarded for tools which offer search functionality based on gene expression in specific tissues or experimental conditions. Regarding the "Functional annotations comparator", "A" refers to an annotation-centred functionality: return the list of homologs corresponding to a given functional annotation. "G" refers to a gene-centred functionality: for a given gene and its homologs, classify the ontology annotations depending on their degree of commonality.

Tools	Dot plot	Trapezoid		Chromo -some painting	Bar track	Genomic contexts centred	Symbolic represen -tation	Homologs browsing of user-defined gene sets	Filters for phylo-genetic profiling	Functional annotations comparator	Target taxonomic kingdoms
		Parallel	Circular								
Absynte (61)						X					Archaea, Bacteria
ACT (62)		X			X						All
AutoGRAPH (63)		X					+				All
BugView (64)	X	X									All
Cinteny (34)		X		X							All
Circos (65)			X		X						All
CoGe (66)		X			X						Plantae
Combo (67)	X	X			X						All
Compam (68)		X									All
Cmap (69)		X									All
CMR (37)	X			X	X			X	AD	A	Archaea, Bacteria
CViT (70)				X							All
GAME synteny viewer (Apollo) (71)		X		X	X						All

EDGAR (72)	X					X			A		Archaea, Bacteria
Ensembl MulticontigView (73)		X			X			X			All
Gbrowse_syn (74)		X			X						All
GeConT (75)						X					Archaea, Bacteria
GeneOrder (76)	X										Archaea, Bacteria
GenomeMatcher (77)	X	X									All
Genomicus (78)					X	X	+				Animalia, Plantae, Protista, Fungi
Gobe (79)		X									All
IMG (80)	X	X			X	X		X	ADE	A	All
Insyght		X				X	++	X	ABC	AG	Archaea, Bacteria
MAGE (18)		X			X			X	AD	A	Archaea, Bacteria
MBGD (81)	X	X			X	X		X		A	Archaea, Bacteria
MCScanX (82)	X	X	X	X							All
MEDEA (www.broadinstitute.org/annotation/medea/)	X	X		X	X				A	A	All
mGSV (83)		X	X		X						All
MicrobesOnline (84)						X		X	AE	A	All
MizBee (85)		X	X	X							All
Narcisse (86)	X	X	X		X						All
OrthoClusterDB (35)				X							All

**Phigs (87)**		**X**								**A**	**All**
**progressiveMauve (88)**		**X**		**X**	**X**						**All**
**PSAT (89)**						**X**			**AD**		**Archaea, Bacteria**
**r2cat (90)**	**X**										**All**
**Sockeye (91)**					**X**				**A**		**All**
**Sybil (92)**		**X**		**X**		**X**		**X**	**AC**	**A**	**Archaea, Bacteria**
**SynBrowse (93)**		**X**			**X**						**All**
**SynteView (94)**					**X**		**+**				**Archaea, Bacteria**
**SynView (95)**		**X**			**X**						**All**
**SyMAP (4)**	**X**	**X**	**X**		**X**			**X**	**A**	**A**	**All**
**VisGenome with CartoonPlus (96)**		**X**					**+**				**All**
**Yeast Gene Order Browser (97)**					**X**		**+**				**Fungi**

References:

4. Soderlund, C., Bomhoff, M. and Nelson, W.M. (2011) SyMAP v3.4: a turnkey synteny system with application to plant genomes. *Nucleic Acids Res*, **39**, e68.

18. Vallenet, D., Labarre, L., Rouy, Z., Barbe, V., Bocs, S., Cruveiller, S., Lajus, A., Pascal, G., Scarpelli, C. and Medigue, C. (2006) MaGe: a microbial genome annotation system supported by synteny results. *Nucleic Acids Res*, **34**, 53-65.

34. Sinha, A.U. and Meller, J. (2007) Cinteny: flexible analysis and visualization of synteny and genome rearrangements in multiple organisms. *BMC Bioinformatics*, **8**, 82.

37. Davidsen, T., Beck, E., Ganapathy, A., Montgomery, R., Zafar, N., Yang, Q., Madupu, R., Goetz, P., Galinsky, K., White, O. *et al.* (2010) The comprehensive microbial resource. *Nucleic Acids Res*, **38**, D340-345.

35. Ng, M.P., Vergara, I.A., Frech, C., Chen, Q., Zeng, X., Pei, J. and Chen, N. (2009) OrthoClusterDB: an online platform for synteny blocks. *BMC Bioinformatics*, **10**, 192.

61. Despalins, A., Marsit, S. and Oberto, J. (2011) Absynte: a web tool to analyze the evolution of orthologous archaeal and bacterial gene clusters. *Bioinformatics*, **27**, 2905-2906.
62. Carver, T.J., Rutherford, K.M., Berriman, M., Rajandream, M.A., Barrell, B.G. and Parkhill, J. (2005) ACT: the Artemis Comparison Tool. *Bioinformatics*, **21**, 3422-3423.
63. Derrien, T., Andre, C., Galibert, F. and Hitte, C. (2007) AutoGRAPH: an interactive web server for automating and visualizing comparative genome maps. *Bioinformatics*, **23**, 498-499.
64. Leader, D.P. (2007) BugView: a tool for genome visualization and comparison. *Methods Mol Biol*, **395**, 109-132.
65. Krzywinski, M., Schein, J., Birol, I., Connors, J., Gascoyne, R., Horsman, D., Jones, S.J. and Marra, M.A. (2009) Circos: an information aesthetic for comparative genomics. *Genome Res*, **19**, 1639-1645.
66. Lyons, E., Pedersen, B., Kane, J., Alam, M., Ming, R., Tang, H., Wang, X., Bowers, J., Paterson, A., Lisch, D. *et al.* (2008) Finding and comparing syntenic regions among Arabidopsis and the outgroups papaya, poplar, and grape: CoGe with rosids. *Plant Physiol*, **148**, 1772-1781.
67. Engels, R., Yu, T., Burge, C., Mesirov, J.P., DeCaprio, D. and Galagan, J.E. (2006) Combo: a whole genome comparative browser. *Bioinformatics*, **22**, 1782-1783.
68. Lee, D., Choi, J.H., Dalkilic, M.M. and Kim, S. (2006) COMPAM :visualization of combining pairwise alignments for multiple genomes. *Bioinformatics*, **22**, 242-244.
69. Youens-Clark, K., Faga, B., Yap, I.V., Stein, L. and Ware, D. (2009) CMap 1.01: a comparative mapping application for the Internet. *Bioinformatics*, **25**, 3040-3042.
70. Cannon, E.K. and Cannon, S.B. (2011) Chromosome visualization tool: a whole genome viewer. *Int J Plant Genomics*, **2011**, 373875.
71. Lewis, S.E., Searle, S.M., Harris, N., Gibson, M., Lyer, V., Richter, J., Wiel, C., Bayraktaroglir, L., Birney, E., Crosby, M.A. *et al.* (2002) Apollo: a sequence annotation editor. *Genome Biol*, **3**, RESEARCH0082.
72. Blom, J., Albaum, S.P., Doppmeier, D., Puhler, A., Vorholter, F.J., Zakrzewski, M. and Goesmann, A. (2009) EDGAR: a software framework for the comparative analysis of prokaryotic genomes. *BMC Bioinformatics*, **10**, 154.
73. Hubbard, T., Andrews, D., Caccamo, M., Cameron, G., Chen, Y., Clamp, M., Clarke, L., Coates, G., Cox, T., Cunningham, F. *et al.* (2005) Ensembl 2005. *Nucleic Acids Res*, **33**, D447-453.
74. McKay, S.J., Vergara, I.A. and Stajich, J.E. (2010) Using the Generic Synteny Browser (GBrowse_syn). *Curr Protoc Bioinformatics*, **Chapter 9**, Unit 9 12.

75. Martinez-Guerrero, C.E., Ciria, R., Abreu-Goodger, C., Moreno-Hagelsieb, G. and Merino, E. (2008) GeConT 2: gene context analysis for orthologous proteins, conserved domains and metabolic pathways. *Nucleic Acids Res*, **36**, W176-180.
76. Mahadevan, P. and Seto, D. (2010) Rapid pair-wise synteny analysis of large bacterial genomes using web-based GeneOrder4.0. *BMC Res Notes*, **3**, 41.
77. Ohtsubo, Y., Ikeda-Ohtsubo, W., Nagata, Y. and Tsuda, M. (2008) GenomeMatcher: a graphical user interface for DNA sequence comparison. *BMC Bioinformatics*, **9**, 376.
78. Louis, A., Muffato, M., and Roest Crollius, H. (2013) Genomicus: five genome browsers for comparative genomics in eukaryota. *Nucleic Acid Res*, **41(D1)**, D700-D705.
79. Pedersen, B.S., Tang, H. and Freeling, M. (2011) Gobe: an interactive, web-based tool for comparative genomic visualization. *Bioinformatics*, **27**, 1015-1016.
80. Markowitz, V.M., Szeto, E., Palaniappan, K., Grechkin, Y., Chu, K., Chen, I.M., Dubchak, I., Anderson, I., Lykidis, A., Mavromatis, K., Ivanova, N.N., Kyrpides, N.C. (2008) The integrated microbial genomes (IMG) system in 2007: data content and analysis tool extensions. *Nucleic Acids Res*, **36** (Database issue), D528-33.
81. Uchiyama, I. (2007) MBGD: a platform for microbial comparative genomics based on the automated construction of orthologous groups. *Nucleic Acids Res*, **35**, D343-346.
82. Wang, Y., Tang, H., Debarry, J.D., Tan, X., Li, J., Wang, X., Lee, T.H., Jin, H., Marler, B., Guo, H. *et al.* (2012) MCScanX: a toolkit for detection and evolutionary analysis of gene synteny and collinearity. *Nucleic Acids Res*, **40**, e49.
83. Revanna, K.V., Munro, D., Gao, A., Chiu, C.C., Pathak, A. and Dong, Q. (2012) A web-based multi-genome synteny viewer for customized data. *BMC Bioinformatics*, **13**, 190.
84. Dehal, P.S., Joachimiak, M.P., Price, M.N., Bates, J.T., Baumohl, J.K., Chivian, D., Friedland, G.D., Huang, K.H., Keller, K., Novichkov, P.S. *et al.* (2010) MicrobesOnline: an integrated portal for comparative and functional genomics. *Nucleic Acids Res*, **38**, D396-400.
85. Meyer, M., Munzner, T. and Pfister, H. (2009) MizBee: a multiscale synteny browser. *IEEE Trans Vis Comput Graph*, **15**, 897-904.
86. Courcelle, E., Beausse, Y., Letort, S., Stahl, O., Fremez, R., Ngom-Bru, C., Gouzy, J. and Faraut, T. (2008) Narcisse: a mirror view of conserved syntenies. *Nucleic Acids Res*, **36**, D485-490.
87. Dehal, P.S. and Boore, J.L. (2006) A phylogenomic gene cluster resource: the Phylogenetically Inferred Groups (PhIGs) database. *BMC Bioinformatics*, **7**, 201.

88. Darling, A.E., Mau, B. and Perna, N.T. (2010) progressiveMauve: multiple genome alignment with gene gain, loss and rearrangement. *PLoS One*, **5**, e11147.
89. Fong, C., Rohmer, L., Radey, M., Wasnick, M. and Brittnacher, M.J. (2008) PSAT: a web tool to compare genomic neighborhoods of multiple prokaryotic genomes. *BMC Bioinformatics*, **9**, 170.
90. Husemann, P. and Stoye, J. (2010) r2cat: synteny plots and comparative assembly. *Bioinformatics*, **26**, 570-571.
91. Montgomery, S.B., Astakhova, T., Bilenky, M., Birney, E., Fu, T., Hassel, M., Melsopp, C., Rak, M., Robertson, A.G., Sleumer, M. *et al.* (2004) Sockeye: a 3D environment for comparative genomics. *Genome Res*, **14**, 956-962.
92. Riley, D.R., Angiuoli, S.V., Crabtree, J., Dunning Hotopp, J.C. and Tettelin, H. (2012) Using Sybil for interactive comparative genomics of microbes on the web. *Bioinformatics*, **28**, 160-166.
93. Pan, X., Stein, L. and Brendel, V. (2005) SynBrowse: a synteny browser for comparative sequence analysis. *Bioinformatics*, **21**, 3461-3468.
94. Lemoine, F., Labedan, B. and Lespinet, O. (2008) SynteBase/SynteView: a tool to visualize gene order conservation in prokaryotic genomes. *BMC Bioinformatics*, **9**, 536.
95. Wang, H., Su, Y., Mackey, A.J., Kraemer, E.T. and Kissinger, J.C. (2006) SynView: a GBrowse-compatible approach to visualizing comparative genome data. *Bioinformatics*, **22**, 2308-2309.
96. Jakubowska, J., Hunt, E. and Chalmers, M. (2010) VisGenome with CartoonPlus: supporting large scale genomic analyses via physical space deformation. *Future Gener Comp Sy*, **26**, 441-454.
97. Byrne, K.P. and Wolfe, K.H. (2006) Visualizing syntenic relationships among the hemiascomycetes with the Yeast Gene Order Browser. *Nucleic Acids Res*, **34**, D452-455.

Supporting Table 2: Pros and cons of the different graphical paradigms used for the visualisation of syntenies and homologies.

Graphical paradigm	Definition	Pros	Cons	Examples of tools that have developed innovative concepts around the paradigm
**Dot plot**	Two genomic sequences define the axes of a graph and the sequence similarity is mapped in this two dimensional coordinate system. Conserved regions appear as diagonal lines (Supplementary Figure 1-A)	• Large conserved regions are easily identifiable.	• Ill-adapted to portray conservation features whose sizes are at discrepancy with the display scale. For example, it is easy to miss small rearrangements when whole genomes are shown.<br>• Ill-adapted to portray multiple comparisons	• SyMAP (4): multiple genomes or elements (chromosomes, plasmids) are represented back to back along both axes therefore creating a grid of two-by-two dot plot comparisons. The efficiency of this technique is nonetheless limited to a few genomes as the individual dot plots become smaller proportionally to the cumulative length of the genomes and it becomes increasingly difficult to visually correlate two distant and small dot plots.<br>• MEDEA (www.broadinstitute.org/annotation/medea): a distinctive colour is assigned to multiple different organisms that are stacked side by side on the y-axis while a unique reference constitutes the x-axis. The analysis however can be hindered when multiple colours pile up on top of each other
**Reference -centred or block tracks**	Observed in most genome browsers, it refers to the mapping of conserved features onto a reference genomic region. The conserved regions of the reference genome appear as blocks in tracks representing multiple stacked up compared organisms. The background of the blocks can be coloured according to synteny or functional properties (Supplementary Fig 1-B)	• Multiple comparisons can be stacked up on top of each other and still remain legible	• One-way view centred on the reference; the genomic context of the compared genomes cannot be shown simultaneously	• MAGE (18) displays up to three homologs within each pairwise comparison tracks to detect gene fusion or fission
**Genomic**	Multiple stacked-up	• Well-adapted to	• Ill-adapted for broadly	

**context-centred**	genomic regions are centred on a reference gene and its homologs (Supplementary Fig 1-C)	compare closely related genomic contexts<br>• Multiple comparisons can be stacked up on top of each other and still remain legible	scattered conserved regions. Only one locus is displayed for each compared genome and only the homologies for the centred reference gene are guaranteed to be shown. Homologs that are not in the vicinity of the centred homolog will not be displayed.	
**Chromosome painting or banded ideograms**	A different colour is allocated to each reference genome or chromosome. Ideograms of compared genomes are displayed with their conserved regions painted according to this palette (Supplementary Figure 1-D).	• Well-adapted for large conserved genomic regions and their reorganisation	• Ill-adapted to visualise small conserved genomic regions	• Sybil (92) uses a variation where a colour-gradient is defined along each reference (Supplementary Figure 1-D).
**Parallel linked track or trapezoid**	Lines are drawn to join homologous regions between two or more stacked-up genomes. The user visualizes the genomic context together with the rearrangements for each comparison (Supplementary Fig 1-E).	• Well-adapted to visualise simple genomic reshaping occurring at a few loci	• Scattered and highly segmented rearrangements result in a tangle of lines that is very difficult to comprehend and interact with	• Circos (65) : genomes are laid out in a circular arrangement. This minimizes the cross-over of lines connecting multiple genomic regions.<br>• SyMAP (4) : uses a 3D approach where a reference stands in the middle and multiple two-by-two comparisons revolve around it. This layout also minimizes the cross-over of lines.
**Symbolic representation**	Symbols of uniform size are used instead of a representation proportional to the genomic sizes. In other words, the scale becomes the annotation events and the metric does no longer	• The display scale needs not to be adjusted to the size of the features of interest<br>• Legibility by human eyes	• Not possible to achieve a genome-wide overview of the conserved regions.	Two approaches have been explored to implement this graphical paradigm:<br>• A parallel linked-track representation where the size of genes is standardized: VisGenome with CartoonPlus (96), AutoGRAPH (63). Although the genes are more easily identified, it does not address the risk of confusion due to the jumble of

	depend on the genomic base pair coordinate system but on the legibility by human eyes.			lines.<br>• Table chart where columns are delimited by genes and rows by compared genomes (Supplementary Figure 1-F). This strategy is used by Genomicus (78), Yeast Gene Order Browser (97), and SynteView (94).

References:

4. Soderlund, C., Bomhoff, M. and Nelson, W.M. (2011) SyMAP v3.4: a turnkey synteny system with application to plant genomes. *Nucleic Acids Res*, **39**, e68.

18. Vallenet, D., Labarre, L., Rouy, Z., Barbe, V., Bocs, S., Cruveiller, S., Lajus, A., Pascal, G., Scarpelli, C. and Medigue, C. (2006) MaGe: a microbial genome annotation system supported by synteny results. *Nucleic Acids Res*, **34**, 53-65.

63. Derrien, T., Andre, C., Galibert, F. and Hitte, C. (2007) AutoGRAPH: an interactive web server for automating and visualizing comparative genome maps. *Bioinformatics*, **23**, 498-499.

65. Krzywinski, M., Schein, J., Birol, I., Connors, J., Gascoyne, R., Horsman, D., Jones, S.J. and Marra, M.A. (2009) Circos: an information aesthetic for comparative genomics. *Genome Res*, **19**, 1639-1645.

78. Louis, A., Muffato, M., and Roest Crollius, H. (2013) Genomicus: five genome browsers for comparative genomics in eukaryota. *Nucleic Acid Res*, **41(D1)**, D700-D705.

92. Riley, D.R., Angiuoli, S.V., Crabtree, J., Dunning Hotopp, J.C. and Tettelin, H. (2012) Using Sybil for interactive comparative genomics of microbes on the web. *Bioinformatics*, **28**, 160-166.

94. Lemoine, F., Labedan, B. and Lespinet, O. (2008) SynteBase/SynteView: a tool to visualize gene order conservation in prokaryotic genomes. *BMC Bioinformatics*, **9**, 536.

96. Jakubowska, J., Hunt, E. and Chalmers, M. (2010) VisGenome with CartoonPlus: supporting large scale genomic analyses via physical space deformation. *Future Gener Comp Sy*, **26**, 441-454.

97. Byrne, K.P. and Wolfe, K.H. (2006) Visualizing syntenic relationships among the hemiascomycetes with the Yeast Gene Order Browser. *Nucleic Acids Res*, **34**, D452-455.

Supporting Table 3. Distribution of the dispensable genes set of V583 in relation to 20 loci of interest regarding horizontal gene transfer. These loci were reported in various studies (42,45,46) (column 3 to 5). The dispensable genes set of V583 is retrieved using Insyght by comparing homologies between strains V583, 62, OG1RF, and Symbioflor1 (column 6 to 12). V583 is a strain from clinical origin, 62 is a commensal isolate from a baby, and OG1RF is a derivative of human Isolate which harbours known virulence traits such as gelatinase (GelE), the adhesin for collagen (Ace) and exhibits virulence in mice. The other two strains are Symbioflor1 which is a probiotic from human origin, and D32 which is isolated from pig faeces. Strain D32 was left aside because its pathogenicity phenotype is not well characterised yet. A “+” in the header refers to the presence of homolog within the designated strains; a “-” refers to the absence of homolog. For example the column V583+ / 62+ / OG1RF+ / Symbioflor1- refers to the gene set from V583 that have homologs with 62 and OG1RF but not in Symbioflor1. For most of the 20 loci (presented as rows), a significant number of genes is retrieved when analysing the overall dispensable gene set. The p-value (Binomial law) is presented in parenthesis to show that the distribution of the dispensable genome is significantly biased toward those loci and is not random. Pp2 was the only prophage that did not appear in our results, which correlates with it being part of the core genome (42). A previous study (47) has found that OG1RF appears to not contain the homologous region to the putative pathogenic island EF_0479-EF_0628; this is consistent with our data as this loci appears to share 81 homologs with strain 62 and only 4 with OG1RF.

Name of loci in V583	Number of genes in loci	Loci reported in Paulsen I.T. (2003) [42]	Loci reported in McBride S.M., figure 5 (2007) [45]	Number of genes from loci reported in Solheim M. (2011) additional file 2 [46]	Number of genes from loci reported in the dispensable genome according to Insyght. The dispensable genome is categorized in different combinations of presence (+) / absence (-) of homologs. Only results with significant e-value (in parenthesis) are presented.						
					V583 +	V583 +	V583 +	V583 +	V583 +	V583 +	V583 +
					62 +	62 -	62 +	62 +	62 -	62 -	62 -
					OG1RF +	OG1RF +	OG1RF -	OG1RF -	OG1RF +	OG1RF -	OG1RF -
					Symbioflor1 -	Symbioflor1 +	Symbioflor1 +	Symbioflor1 -	Symbioflor1 -	Symbioflor1 +	Symbioflor1 -
Entire V583 chromosome	*3111*			*247*	*62*	*30*	*49*	*153*	*36*	*73*	*493*
EF_0121 to EF_0166	45		X	1							**37** (8.7E-16)
pp1 (EF_0303 to EF_0355)	53	X	X	5							**46** (3.1E-20)
Pathogenicity island (EF_0479 to EF_0628)	121	X	X	7			**12** (2.5E-7)	**65** (8.8E-50)			**40** (7.7E-6)

EF_0810 to EF_0818	9								**8** (1.4E-13)		
pp2 (EF_1276 to EF_1293)	18	X	X								
EF_1329 to EF_1346	18			8						**10** (1.8E-11)	
pp3 (EF_1417 to EF_1489)	73	X	X	51							**62** (1.7E26)
EF_1812 to EF_1846	28			13	**11** (1E-11)						
Region efaB5 (EF_1847 to EF_1897)	41	X	X	33	**11** (5.4E-10)			**22** (1.6E-16)			
pp4 (EF_1988 to EF_2043)	46	X	X	21			**11** (1.2E-10)	**26** (6.5E-20)			
pp5 (EF_2084 to EF_2145)	62	X	X								**51** (2.5E-21)
EF_2229 to EF_2239	11				**11** (4.6E-16)						
Region vanB (EF_2240 to EF_2351)	106	X	X	55	**8** (9E-4)						**69** (5.7E-23)
EF_2512 to EF_2546	35		X								**33** (7.5E-16)
pp6 (EF_2798 to EF_2855)	54	X	X	1						**33** (2.5E-38)	
pp7 (EF_2936 to EF_2955)	20	X	X								**18** (5.6E-9)
EF_3099 to EF_3105	7			6							**7** (1.3E-4)
EF_3217 to EF_3227	10			10							**7** (9.9E-4)
Entire V583 plasmid B	*62*			*4*	*0*	*0*	*21*	*21*	*0*	*1*	*5*
EFB0010 to EFB0031	21			4			**19** (1E7)				
EFB0032 to EFB0046	14							**12** (5.1E-4)			

References:

42. Paulsen I.T., Banerjei L., Myers G.S., Nelson K.E., Seshadri R., Read T.D., Fouts D.E., Eisen J.A., Gill S.R., Heidelberg J.F., Tettelin H., Dodson R.J., Umayam L., Brinkac L., Beanan M., Daugherty S., DeBoy R.T., Durkin S., Kolonay J., Madupu R., Nelson W., Vamathevan J., Tran B., Upton J.,

Hansen T., Shetty J., Khouri H., Utterback T., Radune D., Ketchum K.A., Dougherty B.A., and Fraser C.M. (2003) Role of mobile DNA in the evolution of vancomycin-resistant Enterococcus faecalis. *Science*, **299** (5615), 2071-4.

45. McBride S.M., Fischetti V.A., Leblanc D.J., Moellering R.C. Jr, and Gilmore M.S. (2007) Genetic Diversity among Enterococcus faecalis. *PLoS ONE*, **2**(7), e582.

46. Solheim M., Brekke M.C., Snipen L.G., Willems R.J., Nes I.F., Brede D.A. (2011) Comparative genomic analysis reveals significant enrichment of mobile genetic elements and genes encoding surface structure-proteins in hospital-associated clonal complex 2 Enterococcus faecalis. *BMC Microbiol*, 11:3.

47. Bourgogne A., Garsin D.A., Qin X., Singh K.V., Sillanpaa J., Yerrapragada S., Ding Y., Dugan-Rocha S., Buhay C., Shen H., Chen G., Williams G., Muzny D., Maadani A., Fox K.A., Gioia J., Chen L., Shang Y., Arias C.A., Nallapareddy S.R., Zhao M., Prakash V.P., Chowdhury S., Jiang H., Gibbs R.A., Murray B.E., Highlander S.K., Weinstock G.M. (2008) Large scale variation in Enterococcus faecalis illustrated by the genome analysis of strain OG1RF. *Genome Biol*, **9**(7), R110.

Supporting Table 4. Species in the public Insyght database that harbour the conserved synteny EF_1875-EF_1879 / EF_2277-EF_2281 (Reference organism: Enterococcus faecalis strain V583).

Species / strains	NCBI taxon Id	Pathogenicity phenotype
Enterococcus faecalis strain V583 = ATCC 700802	226185	Pathogen Human (Urinary infection, Bacteremia, Endocarditis)
Clostridium difficile strain 630	272563	Pathogen Human (Colitis, Diarrhea, Peritonitis)
Enterococcus faecalis strain 62	936153	Pathogen Human (Nosocomial infection, Endocarditis)
Streptococcus mitis strain B6	365659	Pathogen Human (Endocarditis)
Staphylococcus aureus strain Mu3 = ATCC 700698	418127	Pathogen Human (Mastitis, Nosocomial infection)
Staphylococcus aureus strain Mu50 = ATCC 700699	158878	Pathogen Human (Deep abscesses, Endocarditis, Mastitis, Meningitis, Nosocomial infection, Osteomyelitis, Phlebitis, Pneumonia)
Enterococcus faecalis strain OG1RF	474186	Pathogen Human (Urinary infection, Endocarditis, Bacteremia)
Streptococcus suis strain BM407	568814	Pathogen Human (Arthritis, Endocarditis, Meningitis, Septicemia)
Streptococcus pneumoniae strain ATCC 700669 = Spain 23F-1	561276	Pathogen Human (Pneumonia, Meningitis, Bacteremia)
Streptococcus pneumoniae strain CGSP14	516950	Pathogen Human (Pneumonia)
Streptococcus suis strain 05ZYH33	391295	Pathogen Human (Arthritis, Endocarditis, Meningitis, Septicemia)
Streptococcus pneumoniae strain Taiwan19F-14	487213	Pathogen Human (Pneumonia)
Streptococcus pneumoniae strain P1031	488223	Pathogen Human (Pneumonia)
Staphylococcus aureus strain MRSA ST398 = isolate S0385	523796	Pathogen Human (Endocarditis, Mastitis, Meningitis, Osteomyelitis, Phlebitis, Pneumonia, Septicemia)
Streptococcus gallolyticus strain UCN34	637909	Pathogen Human (Endocarditis)
Streptococcus suis strain SC84	568813	Pathogen Human (Arthritis, Endocarditis, Meningitis, Septicemia)
Listeria monocytogenes strain EGD-e = ATCC BAA-679	169963	Pathogen Human (Listeriosis, food poisonning)
Streptococcus pneumoniae strain G54	512566	Pathogen Human (Pneumonia)
Streptococcus pneumoniae strain Hungary19A-6	487214	Pathogen Human (Pneumonia)
Streptococcus suis strain 98HAH33	391296	Pathogen Human (Arthritis, Endocarditis, Meningitis, Septicemia)
Staphylococcus aureus strain TW20 = 0582	663951	Pathogen Human (Endocarditis, Meningitis, Osteomyelitis, Pneumonia, Septicemia)
Clostridium difficile strain R20291	645463	Pathogen Human (Diarrhea, Colitis, Peritonitis)
Clostridium difficile strain CD196	645462	Pathogen Human (Colitis, Diarrhea, Peritonitis)
Streptococcus agalactiae V strain 2603 V/R = ATCC BAA-611	208435	Pathogen Human (Meningitis, Pneumonia, Septicemia)

Supporting Table 5. Species in the public Insyght database that harbour the conserved synteny V583: EF_2270 - EF_2272 (Reference organism: Enterococcus faecalis strain V583).

Species	NCBI taxon id	Pathogenicity phenotype
Enterococcus faecalis strain V583 = ATCC 700802	226185	Pathogen Human (Urinary infection, Bacteremia, Endocarditis)
Streptococcus agalactiae III strain NEM316	211110	Pathogen Human (Meningitis, septicemia)
Streptococcus pyogenes M1 strain MGAS5005 = ATCC BAA-947	293653	Pathogen Human (Endocarditis, Bone infection, Mastoiditis, Meningitis, Myositis, Necrotizing fasciitis, Otitis, Pharyngitis, Pneumonia, Sinusitis, Tonsillitis)
Streptococcus agalactiae V strain 2603 V/R = ATCC BAA-611	208435	Pathogen Human (Meningitis, Pneumonia, Septicemia)
Streptococcus pyogenes strain Manfredo	160491	Pathogen Human (Rheumatic fever)
Streptococcus dysgalactiae strain GGS_124	486410	Pathogen Human (Septicemia, Meningitis, Endocarditis)
Streptococcus pyogenes strain MGAS10750	370554	Pathogen Human (Rheumatic fever)
Streptococcus pyogenes M1 strain SF370 = ATCC 700294	160490	Pathogen Human (Mastoiditis, Otitis, Pharyngitis, Pneumonia, Rheumatic fever, Sinusitis, Tonsillitis)
Streptococcus pyogenes M6 strain ATCC BAA-946 = MGAS10394	286636	Pathogen Human (Tonsillitis, Rheumatic fever, Pharyngitis)
Streptococcus pyogenes strain NZ131	471876	Pathogen Human (Glomerulonephritis)
Streptococcus pyogenes strain MGAS2096	370553	Pathogen Human (Rheumatic fever,Glomerulonephritis)
Streptococcus pyogenes M3 strain ATCC BAA-595 = MGAS315	198466	Pathogen Human (Rheumatic fever)
Streptococcus pyogenes strain MGAS10270	370552	Pathogen Human (Rheumatic fever)
Streptococcus pyogenes strain MGAS9429	370551	Pathogen Human (Rheumatic fever)
Streptococcus pyogenes M18 strain MGAS8232	186103	Pathogen Human (Rheumatic fever)
Streptococcus pyogenes M28 strain MGAS6180	319701	Pathogen Human (Tonsillitis, Pharyngitis)
Streptococcus pneumoniae strain G54	512566	Pathogen Human (Pneumonia)
Streptococcus pneumoniae strain JJA	488222	Pathogen Human (Pneumonia)
Streptococcus equi strain 4047	553482	Pathogen Horse (Strangles)
Clostridium perfringens strain SM101 = Type A	289380	Pathogen Human (Dysenteria, Enterocolitis, Enterotoxemia, Food poisoning, Gas gangrene)
Clostridium perfringens strain ATCC 13124 = NCTC 8237 = Type A	195103	Pathogen Human (Dysenteria, Enterocolitis, Enterotoxemia, Food poisoning, Gas gangrene)
Streptococcus suis strain SC84	568813	Pathogen Human (Arthritis, Endocarditis, Meningitis, Septicemia)
Streptococcus suis strain 98HAH33	391296	Pathogen Human (Arthritis, Endocarditis, Meningitis, Septicemia)
Streptococcus pneumoniae strain ATCC 700669 = Spain 23F-1	561276	Pathogen Human (Pneumonia, Meningitis, Bacteremia)
Streptococcus pneumoniae strain CGSP14	516950	Pathogen Human (Pneumonia)

Streptococcus pneumoniae strain Hungary19A-6	487214	Pathogen Human (Pneumonia)
Lactobacillus casei strain BL23	543734	Probiotic
Lactobacillus rhamnosus strain Lc 705	568704	Probiotic
Streptococcus pneumoniae strain Taiwan19F-14	487213	Pathogen Human (Pneumonia)
Streptococcus pneumoniae strain ATCC BAA-255 = R6	171101	Pathogen Human (Pneumonia)
Streptococcus pneumoniae strain TIGR4 = ATCC BAA-334	170187	Pathogen Human (Pneumonia, Otitis media, Meningitis)
Streptococcus pneumoniae strain P1031	488223	Pathogen Human (Pneumonia)
Streptococcus equi strain MGCS10565	552526	Pathogen Human (nephritis)
Streptococcus suis strain BM407	568814	Pathogen Human (Arthritis, Endocarditis, Meningitis, Septicemia)
Streptococcus suis strain P1/7	218494	Pathogen Human (Septicemia, Meningitis, Endocarditis, Arthritis)
Streptococcus suis strain 05ZYH33	391295	Pathogen Human (Arthritis, Endocarditis, Meningitis, Septicemia)
Streptococcus suis strain GZ1	423211	Pathogen Human (Pneumonia, Meningitis, Arthritis)
Streptococcus pneumoniae strain D39 = NCTC 7466	373153	Pathogen Human (Pneumonia, Otitis media, Meningitis)
Streptococcus pneumoniae strain 70585	488221	Pathogen Human (Pneumonia)
Lactobacillus casei strain ATCC 334	321967	Probiotic
Streptococcus equi strain H70	40041	Pathogen Horse (Opportunistic infection)
